# Hexanucleotide motifs mediate recruitment of the RNA elimination machinery to silent meiotic genes

**DOI:** 10.1098/rsob.120014

**Published:** 2012-03

**Authors:** Akira Yamashita, Yuichi Shichino, Hirotsugu Tanaka, Edwige Hiriart, Leila Touat-Todeschini, Aurélia Vavasseur, Da-Qiao Ding, Yasushi Hiraoka, André Verdel, Masayuki Yamamoto

**Affiliations:** 1 Department of Biophysics and Biochemistry, Graduate School of Science, University of Tokyo, Hongo, Tokyo 113-0033, Japan; 2 INSERM, U823, Université Joseph Fourier–Grenoble 1, Institut Albert Bonniot, Faculté de Médecine, Domaine de la Merci, La Tronche Cedex, France; 3 Kobe Advanced ICT Research Center, National Institute of Information and Communication Technology, Kobe 651-2492, Japan; 4 Graduate School of Frontier Biosciences, Osaka University, 1-3 Yamadaoka, Suita 565-0871, Japan; 5 Kazusa DNA Research Institute, 2-6-7 Kazusa-kamatari, Kisarazu, Chiba 292-0818, Japan

**Keywords:** mRNA degradation, RNA-binding protein, exosome, meiosis, Mmi1

## Abstract

The selective elimination system blocks the accumulation of meiosis-specific mRNAs during the mitotic cell cycle in fission yeast. These mRNAs harbour a region, the determinant of selective removal (DSR), which is recognized by a YTH-family RNA-binding protein, Mmi1. Mmi1 directs target transcripts to destruction in association with nuclear exosomes. Hence, the interaction between DSR and Mmi1 is crucial to discriminate mitosis from meiosis. Here, we show that Mmi1 interacts with repeats of the hexanucleotide U(U/C)AAAC that are enriched in the DSR. Disruption of this ‘DSR core motif’ in a target mRNA inhibits its elimination. Tandem repeats of the motif can function as an artificial DSR. Mmi1 binds to it *in vitro*. Thus, a core motif cluster is responsible for the DSR activity. Furthermore, certain variant hexanucleotide motifs can augment the function of the DSR core motif. Notably, meiRNA, which composes the nuclear Mei2 dot required to suppress Mmi1 activity during meiosis, carries numerous copies of the core/augmenting motifs on its tail and is indeed degraded by the Mmi1/exosome system, indicating its likely role as decoy bait for Mmi1.

## 2. Introduction

The gene-expression profile differs greatly between mitotic and meiotic cells. In the fission yeast *Schizosaccharomyces pombe*, hundreds of transcripts are newly induced or upregulated during meiosis [1], and various types of post-transcriptional regulation, in addition to transcriptional regulation, are involved in these changes [2–5]. Mmi1, which belongs to the RNA-binding protein family YTH [6], plays a pivotal role in a post-transcriptional event termed selective elimination of meiosis-specific mRNAs [3,7]. Mmi1 recognizes a group of meiosis-specific transcripts that are expressed inappropriately in mitotic cells and removes them in cooperation with nuclear exosomes [3,8]. The target transcripts carry a region known as the DSR (determinant of selective removal), to which Mmi1 binds. If this elimination system does not operate, cells cannot continue robust mitotic proliferation owing to an accumulation of deleterious meiosis-specific transcripts [3].

During meiosis, however, the DSR/Mmi1-dependent elimination system itself becomes deleterious. Mei2, the master regulator of meiosis in fission yeast, plays a key role in circumventing this. In meiotic prophase, Mei2 forms a chromosome-associated dot structure together with non-coding RNA (meiRNA) transcribed from the *sme2* gene, at this gene locus on chromosome II [9–11]. The Mei2 dot sequesters Mmi1 and inhibits its function, so that meiosis-specific mRNAs may be readily and stably expressed [3]. The DSRs in four meiosis-specific genes, namely *mei4*, *rec8*, *ssm4* and *spo5*, were precisely mapped, and the region necessary for proper DSR function was delimited in each case [3]. However, no extensive sequence homology or common features were apparent among these DSRs.

RNA-binding proteins of the YTH family appear to be conserved widely among eukaryotes [6]. Rat YT521-B, the founding member of this family, has been shown to interact with several splicing factors and to alter alternative splice sites in a dose-dependent manner [12,13]. YT521-B was demonstrated to bind to short RNA motifs with high degeneracy [14]. To understand the selective elimination system more profoundly, we set out in our current study to identify the nucleotide motifs that are essential for DSR function using both a computational method known as ‘motif sampling analysis’ [15] and a genetic method involving mutational analysis. Here, we demonstrate that clustering of certain hexanucleotide motifs is responsible for the DSR function. Furthermore, we show that the 3′-tail of the *sme2* transcript is rich in these hexanucleotide motifs, indicating that this RNA may serve as decoy bait to lure Mmi1.

## 3. Results

### 3.1. Identification of a conserved motif U(U/C)AAAC in the determinant of selective removal

To determine whether a common sequence existed in the DSR of different transcripts, we performed motif sampling analysis of this region in *mei4, rec8, ssm4* and *spo5* mRNAs, which we have defined previously [3]. We followed the method described previously by Thijs *et al*. [15]. Although the DSR regions shared no extensive homology among these mRNAs, the hexanucleotide sequences UUAAAC and UCAAAC (electronic supplementary material, figure S1*a*) were identified as the most over-represented motifs among these molecules. As shown in electronic supplementary material, figure S1*b*, the *mei4*, *rec8*, *ssm4* and *spo5* DSRs carried seven, four, two and five copies of the DSR core motif U(U/C)AAAC in the respective DSR regions assigned previously to them (orange and yellow arrowheads). Additional copies of this core motif were found to be scattered throughout the mRNA sequences (black and grey arrowheads in electronic supplementary material, figure S1*b*), which might also contribute to DSR function. It was noted that the *rec8* mRNA carried a second cluster of this core motif within its ORF, which we had not identified in our previous analysis. We confirmed that this region could confer the DSR activity, although it was weaker than the original one (data not shown). More precise arrangements of the core motif in each DSR are depicted in electronic supplementary material, figure S1*c*.

### 3.2. Substitution mutations affecting the core motifs block the function of determinant of selective removal

In parallel with the aforementioned computer analysis, we wished to pinpoint the region that was critical for *spo5* DSR function. We performed deletion analysis of a previously described reporter gene [3] comprising the constitutive *adh1* promoter, the *GFP* ORF and the *spo5* DSR. This chimeric gene generated few *GFP*-*spo5DSR* transcripts in mitotically growing cells (electronic supplementary material, figure S2*a*, lane 3), whereas a control construct carrying no DSR region produced *GFP* transcripts abundantly (lane 1). However, in cells undergoing meiosis via the function of active Mei2 [9], the chimeric gene generated considerable levels of *GFP*-*spo5DSR* transcripts (lane 4). We introduced a series of deletions into the *spo5* DSR region and found that removal of 21 nucleotides between positions 1778 and 1798 (electronic supplementary material, figure S1) rendered it non-functional (data not shown). We next introduced arbitrary substitutions into this region. One of these mutations, which we designated *spo5DSR-M10* (electronic supplementary material, figure S2*b*), markedly impaired the DSR activity, as a result of which the mitotic cells accumulated *GFP*-*spo5DSR-M10* transcripts, although at a slightly lower abundance than meiotic cells (electronic supplementary material, figure S2*a*, lane 5 versus lane 6). When matched with the DSR core motif described above, the *M10* mutation was found to involve the complete loss of two contiguous copies of this motif (electronic supplementary material, figure S2*b*).

**Figure 1.**
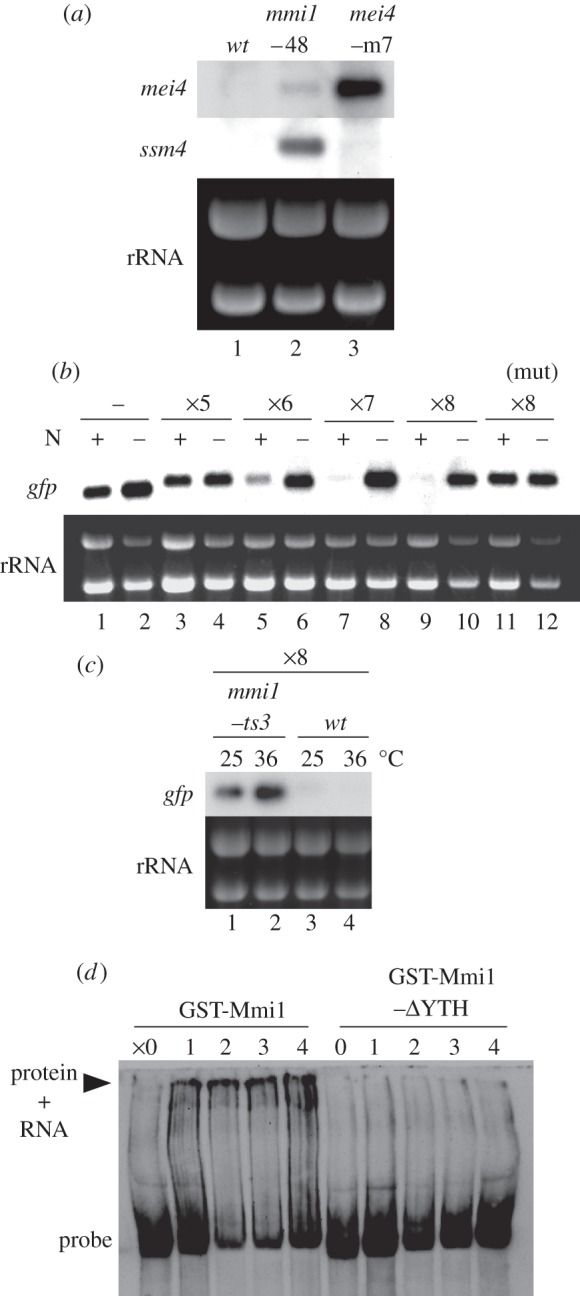
The DSR core motif is central to DSR function. (*a*) Expression of *mei4* and *ssm4* mRNAs in the *mei4-m7* cells in which DSR core motifs in the *mei4* DSR were abrogated. Transcripts of *mei4* and *ssm4* were analysed by northern blotting in JY450 (wild-type, lane 1), JT221 (*mmi1-48*, lane 2) and JT925 (*mei4-m7*, lane 3). rRNAs stained with ethidium bromide are shown in the bottom panel as loading controls. (*b*) Tandem repeats of the DSR core motif can reconstitute DSR function. Expression of *GFP* mRNA was examined from the reporter chimeric gene with no copy of the TTAAAC core motif (JT634, lanes 1 and 2), with five copies (JT629, lanes 3 and 4), with six copies (JT630, lanes 5 and 6), with seven copies (JT631, lanes 7 and 8), with eight copies (JT632, lanes 9 and 10) or with eight copies of a mutated motif GTAAAC (JT633, lanes 11 and 12). +N lanes represent cells growing mitotically, whereas −N lanes represent cells undergoing meiosis, starved of nitrogen for 4 h. rRNAs stained with ethidium bromide are shown in the bottom panel as loading controls. (*c*) Expression of *GFP* mRNA from the reporter chimeric gene with eight copies of the TTAAAC core motif in JT923 (*mmi1-ts3*, lanes 1 and 2) and JT916 (wild-type, lanes 3 and 4) cells. Each strain was grown at 25°C and sampled before (lanes 1 and 3) and after (lanes 2 and 4) the shift to 36°C for 2 h. rRNAs stained with ethidium bromide are shown in the bottom panel as loading controls. (*d*) Electrophoretic mobility shift assay (EMSA) for the binding of GST-Mmi1 and GST-Mmi1 lacking the YTH domain (*Δ*YTH) to 1–4 tandem repeats of the DSR core motif (UUAAAC) fused to the *GFP* ORF transcript.

We next prepared a mutant strain in which all of the seven DSR core motifs in the *mei4* DSR were mutated without affecting the protein coding capacity (see §5). The resultant *mei4-m7* strain showed a weak growth defect (data not shown). In this strain, *mei4* transcripts, but not *ssm4* transcripts, were visibly expressed under the nutrient conditions, even at a greater level than in the hypomorphic *mmi1-48* mutant (figure 1*a*) [3]. This confirms that the core motif is central to the functional activity of the DSR region, and also explains the observed growth defect of the strain, because ectopic expression of *mei4* is deleterious for vegetative cell growth [3,16,17].

### 3.3. Tandem repeats of the core motif exhibit determinant of selective removal activity

The abovementioned observations led us to examine whether repeats of the DSR core motif exhibited DSR functional activity on their own. We constructed a set of yeast strains containing a chimeric gene composed of the *adh1* promoter, the *GFP* ORF and a DNA fragment carrying 1–8 copies of the DSR core motif (TTAAAC). The repeat motif sequences were separated by intervals containing six-base restriction enzyme recognition sequences (see §5). The quantity of transcripts generated from the chimeric gene in each *S. pombe* strain was measured in both mitotic and meiotic cells. As shown in figure 1*b*, the chimeric gene carrying up to five copies of TTAAAC exerted no significant DSR effect (lanes 3 and 4). In contrast, the constructs carrying more than five copies of TTAAAC failed to accumulate transcripts in mitotic cells, suggesting that a tandem array of six or more copies of the core motif can function as a DSR (figure 1*b*, lanes 5, 7 and 9). A control strain carrying eight copies of a mutated motif GTAAAC expressed abundant transcripts in mitotic cells (lane 11). Transcripts of the construct carrying eight copies of TTAAAC were accumulated visibly when the activity of Mmi1 was weakened by the temperature-sensitive mutation (figure 1*c*), indicating that they are indeed eliminated through the Mmi1-dependent degradation machinery.

### 3.4. Physical interaction of the determinant of selective removal core motif with Mmi1

We previously showed that Mmi1 could directly bind to the DSR of *mei4, rec8, ssm4* and *spo5* transcripts *in vitro* [3]. We thus tested in our current study whether the core motif could bind Mmi1 using a non-radioisotope electrophoretic mobility shift assay (EMSA; see §5). A single copy of the DSR core motif, fused to the *GFP* ORF transcript, could be trapped considerably by Mmi1, and the binding was more effective if the transcript carried multiple copies of the motif, whereas Mmi1 lacking its YTH domain did not bind to it (figure 1*d* and electronic supplementary material, figure S3*a*). This indicates that the core motif is indeed a pivotal element of the DSR during recognition by the YTH domain. If a mutated motif (GUAAAC) was used, the binding was not detected, even with a transcript carrying eight copies of it (electronic supplementary material, figure S3*a*).

We next quantitatively determined the affinity between Mmi1 and four tandem repeats of the DSR core motif fused to the *GFP* ORF, by titrating the amount of Mmi1, as was done with YT521-B previously [14] (see §5). The *K*_d_ value was calculated to be 65 nM (electronic supplementary material, figure S3*b*). This affinity was 400 times greater than that of YT521-B for its targets (26 µM). This big difference of affinity might reflect the distinct function of the two proteins, YT521-B being a splicing regulator and Mmi1 being an inducer of mRNA degradation.

### 3.5. meiRNA carries ample copies of the motif on its tail

**Figure 2.**
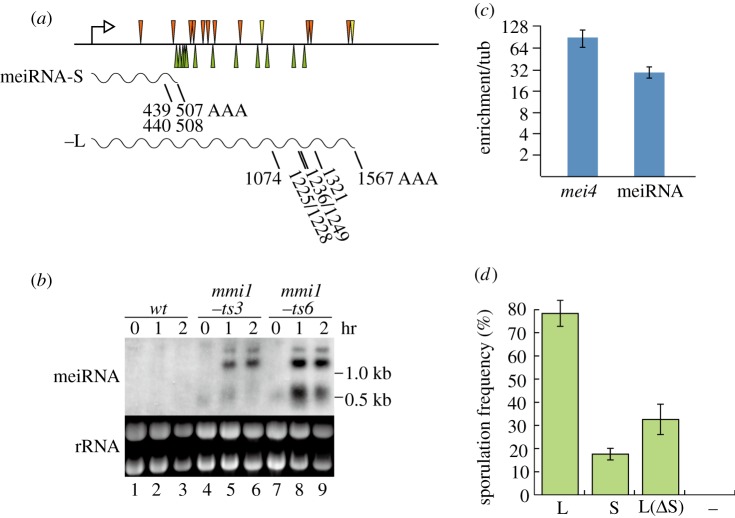
meiRNA is a target of Mmi1. (*a*) Location of the DSR core motif within meiRNA. The UUAAAC sequence is indicated by an orange arrowhead and UCAAAC is indicated by a yellow arrowhead. The augmenting motifs (UAAAAC, UGAAAC and GUAAAC) are indicated by green arrowheads. We determined the precise 3′-ends of long transcripts (meiRNA-L) by RT–PCR. (*b*) Expression of meiRNA encoded by the *sme2* gene in the *mmi1* mutants. Transcripts of the *sme2* gene (meiRNA) were analysed by northern blotting in cells of JY450 (wild-type, lanes 1–3), JV579 (*mmi1-ts3*, lanes 4–6) and JV582 (*mmi1-ts6*, lanes 7–9). Each strain was grown at 25°C, shifted to 36°C and sampled at the indicated time points. (*c*) Association of meiRNA with Mmi1. RNA-IP was performed for meiRNA and a positive control *mei4* mRNA using anti-Mmi1 antibodies. Enrichment over *tub1* mRNA is displayed for each. Error bars indicate standard deviations for four independent measurements. (*d*) Recovery of sporulation in the *sme2Δ* mutant by artificial expression of meiRNA-L, meiRNA-S and meiRNA-L without the meiRNA-S portion. Derivatives of the *sme2* gene encoding either meiRNA-L (L; 1-1562), its 5′ portion (S; 1–508) or its 3′ portion (L(*Δ*S); 509–1562) were expressed from the vector pREP1 in homothallic *sme2Δ* cells (JT926), and their sporulation frequency was measured after incubation on SSA medium at 30°C for 3 days. Error bars indicate standard deviations (three measurements for each; total *n* > 800). The control transformant carrying pREP1 is indicated by (–).

Given the above findings, we performed a genome-wide search for genes carrying repeats of the DSR core motif. This resulted in the identification of a number of candidate genes, most of which turned out to be meiosis-specific. They include *mug4* (five copies), *mug8* (seven copies), *mug10* (six copies) and *mug45* (10 copies), and their ectopic expression in the *mmi1-ts* mutants was indeed confirmed by northern blotting (data not shown). One highly inspiring outcome of this search, among others, was that the 3′-end region of the *sme2* gene, which encodes meiRNA required to block the selective removal of DSR-containing mRNAs during meiosis [3,18], is abundantly occupied by the motif (figure 2*a*).

The *sme2Δ* cells, which cannot develop the Mei2 dot structure to sequester Mmi1, eventually arrest prior to meiosis I [3,10]. In our original characterization, we noticed that meiRNA was transcribed from the *sme2* gene mainly as short polyadenylated transcripts of about 0.5 kb in length (439/440 and 507/508 nucleotides excluding poly(A)). We also detected minor doublet bands of about 1.2 kb in meiotic cells, but we interpreted them as probable read-through products that overlapped with the 0.5 kb transcripts [18]. The results described above urged us to recharacterize *sme2* transcripts. In the following analyses, we refer to the short canonical meiRNA as meiRNA-S and the long *sme2* transcripts collectively as meiRNA-L.

We set out to determine polyadenylation sites of meiRNA-L, recovered from meiotic cells, by 3′-RACE (rapid amplification of cDNA ends). As summarized in figure 2*a*, this analysis revealed that meiRNA-L is polyadenylated at seven sites at least, which correspond to the transcript length of 1.1–1.6 kb. These meiRNA-L species carried 9–13 copies of the DSR core motif (figure 2*a*). We have previously shown that meiRNA-S has a moderate affinity for Mmi1 and is likely to sequester this protein in cooperation with Mei2, which also has an affinity for Mmi1 [3]. Expression of meiRNA-L was detectable only in cells undergoing meiosis [18], like DSR-containing meiotic transcripts, but meiRNA itself was dispensable for the progression of meiosis once the activity of Mmi1 was reduced [3]. These observations strongly suggested that meiRNA-L might function as a decoy bait to lure Mmi1. In agreement with this idea, expression of meiRNA-L was elevated enormously in the *mmi1-ts* mutants shifted to the restrictive temperature on nutrient medium: transcripts of approximately 1.2 and 1.5 kb in length were detected clearly, in addition to 0.5 kb meiRNA-S (figure 2*b*).

We then tested whether Mmi1 could bind to meiRNA in fission yeast cells by RNA immunoprecipitation (RNA-IP) experiments, as described in §5. Similar to the positive control *mei4* mRNA, meiRNA was enriched in the immunoprecipitated Mmi1 complex (figure 2*c*). These results altogether indicate that meiRNA-L is a substrate of the Mmi1-dependent selective elimination, which may compete with DSR-containing meiosis-specific mRNAs. The significance of this competitor function of meiRNA in the induction of meiosis was supported by the observation that expression of meiRNA-L missing the meiRNA-S sequence could partially suppress *sme2Δ* (figure 2*d*).

### 3.6. Expression of artificial bait can suppress loss of meiRNA

The role of meiRNA as a competitor of the DSR-containing transcripts for selective degradation well explains our previous observation that the high expression of any DSR-containing RNA could suppress meiotic deficiency and recover sporulation in the *sme2Δ* strain to some extent [3]. Excess DSR-containing RNA is likely to lure Mmi1 during meiotic prophase, as meiRNA-L does, and hence to alleviate degradation of meiosis-specific mRNAs. To confirm this possibility, we mutated core motifs in the *mei4* DSR and *rec8* DSR, carrying seven and four copies of the motif, respectively, and tested their *sme2Δ* suppression activity (electronic supplementary material, figure S4). Overexpression of the DSR sequence fused to the *GFP* ORF from the strong *nmt1* promoter suppressed the *sme2Δ* phenotype efficiently (*mei4* DSR, about 50%; *rec8* DSR, about 25%; electronic supplementary material, figure S4*a*,*b*). We then introduced mutations to motifs 4, 5 and 6 of *mei4* DSR and 1–4 of *rec8* DSR (see §5). Mutations that damaged a single motif in the *mei4* DSR decreased the suppression efficiency to a varying extent, with the M6 mutation showing the largest decrease (electronic supplementary material, figure S4*a*). Thus, each motif appeared to contribute to the suppression activity to a different degree, depending on its position in the DSR. Significantly, a combination of these mutations further reduced the suppression activity, confirming that the DSR core motif was a key determinant of the suppression efficiency. We obtained the same conclusions from analyses of the *rec8* DSR (electronic supplementary material, figure S4*b*).

### 3.7. Evaluation of the determinant of selective removal activity of various hexanucleotide sequences

**Figure 3.**
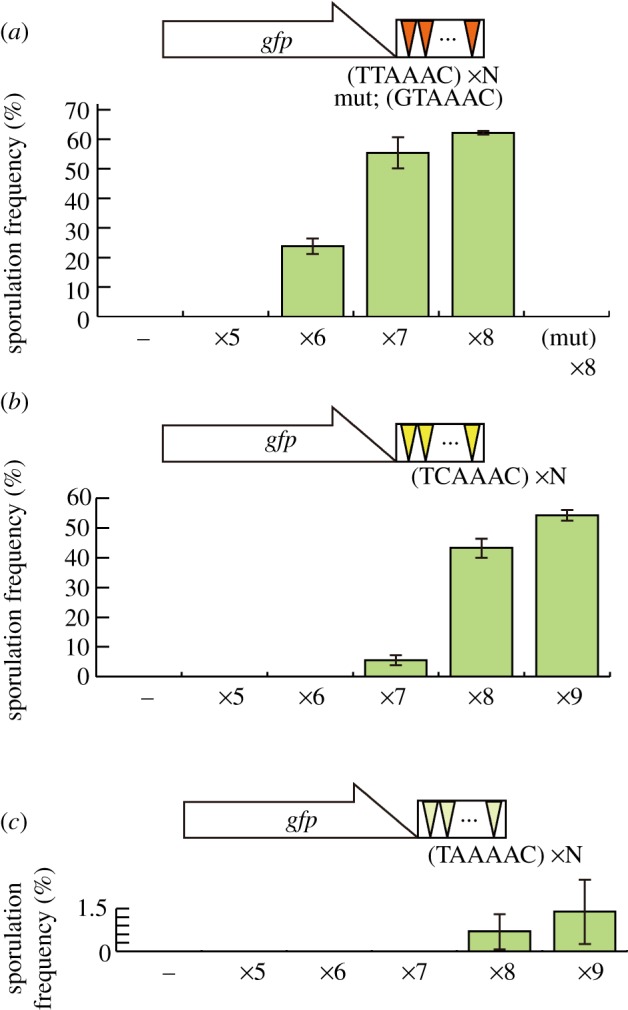
Evaluation of the DSR activity of hexanucleotide repeats. (*a*) Sporulation frequency of JZ464 (*sme2Δ*) cells expressing *GFP* fused with tandem repeats of the DSR core motif (TTAAAC) or a mutant form (GTAAAC) from the multi-copy plasmid pRGT1. The number of repeats in each construct is depicted by ×N. The control strain carrying pRGT1 is indicated by (–). The sporulation frequency was determined by microscopic observations after incubation on SSA medium at 30°C for 3 days. Error bars indicate standard deviations (three measurements for each; total *n* > 400). (*b*) Sporulation frequency of JZ464 cells expressing *GFP* fused with tandem repeats of the DSR core motif (TCAAAC), assayed as in (*a*). (*c*) Sporulation frequency of JZ464 cells expressing *GFP* fused with tandem repeats of a variant motif (TAAAAC), assayed as in (*a*).

Taking advantage of the relative ease of the *sme2Δ* suppression assay, we evaluated the DSR activity of various hexanucleotide sequences. Transcripts carrying more than five copies of the core motif UUAAAC suppressed the meiotic arrest of the *sme2Δ* mutant (figure 3*a*), in good agreement with the results obtained by the degradation assay (figure 1*b*). The suppression efficiency increased in a copy number-dependent manner (figure 3*a*). If the motif was altered to GUAAAC, the suppression was abrogated.

The DSR activity of repeats of UCAAAC, UAAAAC and UGAAAC was similarly evaluated. UCAAAC, which we defined as the other core motif sequence, revealed weak DSR activity in seven copies, and fairly strong activity when eight or more copies were aligned (figure 3*b*). UCAAAC is thus slightly less effective than UUAAAC. Two variant sequences, namely UAAAAC and UGAAAC, are also frequently seen in the DSR region, as typically illustrated for meiRNA in figure 2*a*. However, UAAAAC revealed very low DSR activity, if any, when eight or nine copies were aligned (figure 3*c*). UGAAAC showed no detectable DSR activity even when 10 copies were aligned (data not shown). Therefore, we tentatively discriminate these sequences from the core motif.

### 3.8. Hexanucleotides that augment the function of the determinant of selective removal core motif

**Figure 4.**
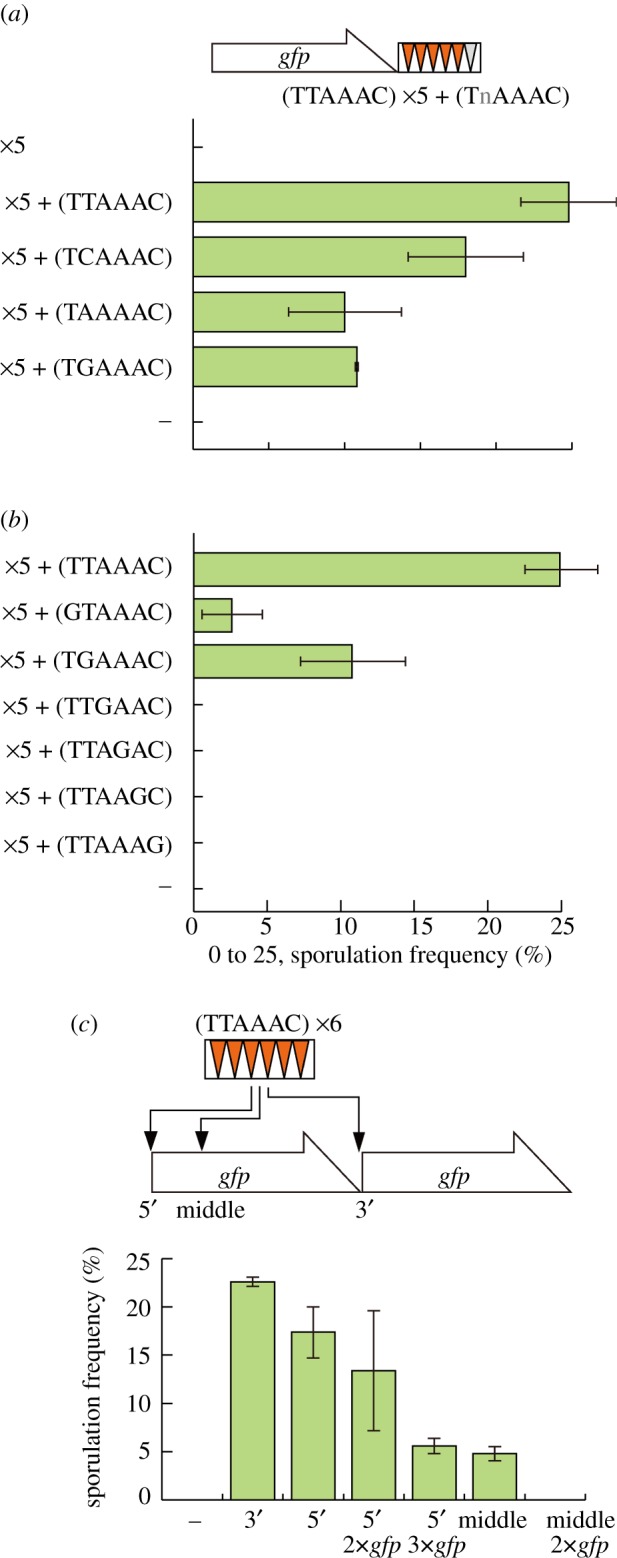
DSR-augmenting motifs. (*a*) Sporulation frequency of JZ464 (*sme2Δ*) expressing *GFP* fused with an array of five copies of the DSR core motif (TTAAAC) plus one copy of a variant motif from a multi-copy plasmid pRGT1. Variants carrying an alteration at the second position were examined. The control strain carrying no additional variant is indicated by ×5, and that carrying only pRGT1 is indicated by (–). Error bars indicate standard deviations (three measurements for each; total *n* > 400). (*b*) Variants carrying G in the first to sixth positions were examined as in (*a*). The control strain carrying pRGT1 is indicated by (–). (*c*) Sporulation frequency of JZ464 cells expressing *GFP* fused with six copies of the DSR core motif (TTAAAC) at its 5′- or 3′-ends, or in the midst of the ORF. 2×*gfp* and 3×*gfp* represent constructs that have one or two extra copies of the *GFP* ORF at the 3′-end, respectively. Error bars indicate standard deviations (three measurements for each; total *n* > 400).

Even though unable to exert significant DSR activity by themselves, the frequent occurrence of UAAAAC and UGAAAC in the DSR regions implied that they may somehow contribute to the DSR function. Natural DSRs often carry less than six copies of the core motif. We thus tested whether these hexanucleotides might augment the function of the core motif, using the test system described below. We first integrated five copies of the DSR core motif (TTAAAC), which by themselves did not confer sufficient DSR activity, at the end of the *GFP* ORF on pRGT1. We next placed a single hexanucleotide sequence to be tested after these five repeats. The DSR activity of the resulting composite array was then measured via the *sme2Δ* suppression assay. If the last copy was TTAAAC (i.e. if there were six copies of TTAAAC) the array could function as an effective DSR (figure 4*a*; also see figure 3*a*). The addition of TCAAAC also generated considerable DSR activity, as expected. Notably, TAAAAC and TGAAAC could also recover DSR activity significantly (figure 4*a*).

Variants in which the third, fourth, fifth or sixth position of the TTAAAC motif was substituted by G exhibited no *sme2Δ* suppression when they were linked to the 5×TTAAAC array, indicating that these altered motifs possessed no DSR-enhancing activity (figure 4*b*). Interestingly, however, a first position G substitution (GTAAAC) could induce weak suppression activity (figure 4*b*), although tandem repeats of eight copies of GUAAAC could not reconstitute DSR function (figures 1*b* and 3*a*).

The above observations indicated that the UAAAAC, UGAAAC and GUAAAC sequences could contribute to the DSR activity if combined with the canonical core motif U(U/C)AAAC. We thus refer to these sequences as ‘DSR-augmenting motifs’. Given these results, we revisited the sequences of the *mei4, rec8, ssm4* and *spo5* genes, and determined the number of both core and augmenting motifs in each respective DSR region (table 1). The *ssm4* DSR carries only two copies of the core motif but harbours five copies of the augmenting motif (hence seven copies in total). In contrast, the *spo5* DSR contains five copies of the core motif but only one copy of the augmenting motif. There are 13 copies of the core motif and 12 copies of the augmenting motif on the longest meiRNA-L (table 1).

**Table 1.** The copy number of DSR core/augmenting motifs in each DSR.

	core motif		augmenting motif			total
	UUAAAC	UCAAAC	UAAAAC	UGAAAC	GUAAAC	
*mei4* DSR	3	4	0	1	0	8
*rec8* DSR	4	0	1	1	0	6
*ssm4* DSR	2	0	1	4	0	7
*spo5* DSR	2	3	1	0	0	6
meiRNA-L	11	2	5	7	0	25

### 3.9. Overall positional effects upon determinant of selective removal activity

We investigated whether the activity of a DSR might be dependent on its positioning within the target transcript. We placed an artificial DSR containing six copies of the core motif (TTAAAC) at various positions within pRGT1: immediately after the *GFP* ORF (3′), just before the first codon (5′) or in the midst of the ORF (middle; 167 bp downstream of the first codon; figure 4*c*). The DSR activity levels were again estimated by measuring the suppression of the *sme2Δ* phenotype. As shown in figure 4*c*, the 3′ insertion was the most effective in this regard, followed by the 5′ insertion, whereas the middle insertion was found to be the least effective. When the distance between the 5′ positioned DSR and the tail of the transcript was extended by insertion of one to two more copies of the *GFP* ORF, the suppression efficiency dropped with the increasing distance (figure 4*c*; 5′ 2×*gfp* and 3×*gfp*). The middle DSR also appeared to lose activity if another copy of the *GFP* ORF was inserted (figure 4*c*; middle 2×*gfp*).

The above results may indicate that, in general, a DSR region is more effective when positioned closer to the 3′-end of a transcript, as long as the DNA sequence surrounding it is identical. However, it is also true that the activity of a DSR is much affected by the context in which it is situated.

## 4. Discussion

In our present study, we establish that two hexanucleotide sequences, UUAAAC and UCAAAC, represent the DSR core motif, and play a principal role in exerting DSR activity and function. Our data also show that three further hexanucleotide sequences, UAAAAC, UGAAAC and GUAAAC, are DSR-augmenting motifs that play an assisting functional role, although GUAAAC does not seem to be a preferred choice in nature (table 1). Our analyses have suggested that the cooperation of six or more copies of the core/augmenting motifs is essential to yield full DSR function *in vivo*. A single copy of UUAAAC cannot do so, although it does bind Mmi1 effectively *in vitro*. As the emergence of a single hexanucleotide sequence is not a statistically rare event, it seems likely that the cell has developed additional regulatory processes that restrict DSR activity to a substantial number of repeats of these sequences.

It has been shown recently that Mmi1 induces facultative heterochromatin formation at the *mei4* and *ssm4* loci [19]. However, heterochromatin formation has not been detected at the *rec8* and *spo5* loci, although all four loci encode the targets of Mmi1. Red1, a component of the DSR/Mmi1-dependent elimination system [20], might have a role in discriminating these loci, because it is recruited only to the former and is essential for the assembly of heterochromatin at these loci [19]. Therefore, it is plausible that certain sequence motifs, in addition to the hexanucleotide motifs, may also contribute to the DSR function and give individual traits to each DSR.

One factor that may influence DSR activity but has not been fully explored is the space between neighbouring copies of the core motif. As shown in electronic supplementary material, figure S1*c*, this region is mostly 30–35 bases in length in naturally occurring DSRs, but is also variable. For example, in the *mei4* DSR region, the shortest spacer region is 10 bases and the longest is 57 bases. Moreover, the *spo5* DSR contains two core motif copies that are contiguous. We set the spacer region at 6 bases in our current analysis of artificial DSRs as this enabled ease of manipulation, and the obtained results suggested that constructed DSRs are functionally comparable with naturally occurring DSRs. Hence, the spacing of the DSR core/augmenting motifs may not be critically important for activity, but it remains to be seen how this affects the function of the entire array of motifs.

In a previous study from our laboratory [3], we defined some of the DSR regions within the ORFs of specific genes, which were hundreds of bases in length. This was surprising to us at the time as it was uncertain how these regions within certain genes would not compromise their coding capacity. Given that we have now mapped the essential element underlying DSR activity to a six-base motif, this appears not to be an issue any longer. UYAAAC can be translated in three reading frames, and an analysis of all of the ‘coding’ DSR core motifs shown in electronic supplementary material, figure S1*b* indicates that the core motif is most frequently translated as codons UYA-AAC (15 of 26), followed by UY-AAA-C (9 of 26). U-YAA-AC was found to be relatively rare, but it is unclear if this has any significance as UAA is a stop codon. Taken together, we suspect that it may not be extremely difficult for a useful coding sequence to exist that is strewn with DSR core/augmenting motifs. The use of the augmenting motifs, which are less potent than the core motif, may be rationalized by the selective pressure for certain amino acid sequences in the gene product.

Our analysis of the DSR core/augmenting motifs led to the finding that meiRNA is a target of Mmi1. We have shown that meiRNA and its binding partner Mei2 form an intranuclear dot structure during meiotic prophase, and sequester Mmi1 so that meiotic mRNAs carrying DSR may be stably expressed [3,10]. The short transcripts from *sme2*, namely meiRNA-S, appear to be able to fulfil this function. Given the remarkable structure of meiRNA-L, however, it is now evident that competition between this RNA and DSR-containing mRNAs as substrates for the Mmi1-dependent destruction system also contributes substantially to the suppression of the Mmi1 function during meiosis. How these two kinds of mechanisms are mutually integrated remains currently elusive. So far, in our analysis all the detected transcripts from *sme2* appear to be polyadenylated at the 3′-end. While this is consistent with previous observations that the Mmi1/exosome-dependent RNA degradation is closely associated with conventional polyadenylation [8,21], it is puzzling whether each meiRNA species is transcribed as a distinct polyadenylated RNA; alternatively, meiRNA-S may represent digestion products of meiRNA-L, which somehow undergo repolyadenylation. How highly expressed meiRNA lures Mmi1 to the nuclear dot structure and dampens it offers a challenging question.

## 5. Material and methods

### 5.1. Fission yeast strains, genetic analysis and growth media

The *S. pombe* strains used in this study are listed in table 2. We constructed strains JT629, JT630, JT631, JT632, JT633 and JT634 by inserting a chimeric gene carrying the *adh* promoter, the *GFP* ORF and a varying number of the DSR core motif or its variant at the *lys1* locus on chromosome I. JT925 was constructed by transforming a *mei4::ura4^+^* strain with a mutated *mei4* fragment in which seven DSR core motifs were altered without affecting the protein-coding capacity (M1, AGCAAT; M2, TAAAGC; M3, CTGAAT; M4, AGCAAT; M5, CTGAAT; M6, TGAAGC; M7, AGCAAT). The general genetic procedures used for the analyses of the *S. pombe* strains have been previously described [22]. Yeast transformations were performed using a lithium acetate method [23]. Growth medium included complete medium YE, minimal medium SD and MM [24] and synthetic sporulation medium SSA [25].

**Table 2.** *Schizosaccharomyces pombe* strains used in this study.

strain	genotype
JT219	*h^−^ mmi1-48-kanR ade6-216 leu1*
JT221	*h^90^ mmi1-48-kanR ade6-216 leu1*
JT629	*h^+^/h^−^ lys1^+^/lys1::adh-GFP-(5xDSR core)-KanR ade6-M216/ade6-M210 leu1/leu1*
JT630	*h^+^/h^−^ lys1^+^/lys1::adh-GFP-(6xDSR core)-KanR ade6-M216/ade6-M210 leu1/leu1*
JT631	*h^+^/h^−^ lys1^+^/lys1::adh-GFP-(7xDSR core)-KanR ade6-M216/ade6-M210 leu1/leu1*
JT632	*h^+^/h^−^ lys1^+^/lys1::adh-GFP-(8xDSR core)-KanR ade6-M216/ade6-M210 leu1/leu1*
JT633	*h^+^/h^−^ lys1^+^/lys1/lys1::adh-GFP-(8xmutated DSR core)-KanR ade6-M216/ade6-M210 leu1/leu1*
JT634	*h^+^/h^−^ lys1^+^/lys1/lys1::adh-GFP-KanR ade6-M216/ade6-M210 leu1/leu1*
JT916	*h^−^ lys1::adh-GFP-(8xDSR core)-KanR ade6-M216 leu1*
JT923	*h^−^ lys1::adh-GFP-(8xDSR core)-KanR mmi1-ts3-bsdR ade6-M216 leu1*
JT925	*h^90^ mei4-m7 ade6-M216 leu1*
JT926	*h^90^ sme2(1-1065):: ura4^+^ ade6-M216 leu1 ura4-D18*
JV558	*h^−^ mmi1-kanR ade6-M216 leu1*
JV564	*h^−^ mmi1-ts3-kanR ade6-M216 leu1*
JV567	*h^−^ mmi1-ts6-kanR ade6-M216 leu1*
JV579	*h^90^ mmi1-ts3-kanR ade6-M216 leu1*
JV582	*h^90^ mmi1-ts6-kanR ade6-M216 leu1*
JX383	*h^−^ leu1::nmt41-mei2-SATA-ura4^+^ ade6-M210 ura4-D18*
JY362	*h^+^/h^−^ ade6-M216/ade6-M210 leu1/leu1*
JY450	*h^90^ ade6-M216 leu1*
JZ464	*h^90^ sme2::ura4^+^ ade6-M216 leu1 ura4-D18*

### 5.2. Plasmid construction

The DSR regions of the *mei4* and *rec8* genes were cloned into a *GFP*-fusion vector pRGT1, and that of *spo5* was cloned into pAGT1. pRGT1 is a derivative of the *S. pombe* expression vector pREP1 [26], which harbours the ORF of a mutant version of GFP (Ser65-Thr). pAGT1 is a variant of pRGT1 in which the inducible *nmt1* promoter is replaced by the constitutive *adh1* promoter. The introduction of mutations into the DSR core motif (M1–M7 in *mei4* DSR shown above; and m1, ACTAGT; m2, CCATGG; m3, AGATCT; and m4, CATATG in *rec8* DSR) was achieved either with a standard mutagenesis protocol [27] or as indicated in the instructions for the PrimeSTAR Mutagenesis Basal Kit (Takara Bio). Plasmids carrying tandem repeats of the DSR core motifs were constructed using the oligonucleotides 5′-GGATCC*TTAAAC*AGATCT-3′ and 5′-GGATCC*TTAAAC*GAATTC*TTAAAC*AGATCT-3′ (the core motif is italicized). These molecules were sequentially inserted into pRGT1. To construct a mutant version of the DSR core motif, the TTAAAC sequence in these oligonucleotides was changed to GTAAAC.

### 5.3. Northern and western blotting analysis

Northern blotting analysis was performed as described previously [28] using a DNA probe for the *GFP* sequence or for the gene indicated. Ten micrograms of total cellular RNA was used for each sample. Western blotting analysis was performed using a general method with anti-Mei2 antibodies.

### 5.4. Non-radioisotope electrophoretic mobility shift assay

Digoxigenin (DIG)-labelled RNA was prepared from PCR products according to the manufacturer's instructions (Roche). The RNA binding reaction was performed using 0.4 nM of DIG-labelled RNA and 40 nM of bacterially purified glutathione *S*-transferase (GST)-Mmi1 or GST-Mmi1 lacking the YTH domain (*Δ*YTH; carrying residues 1–292) in 10 µl of a modified KNET buffer: 20 mM KCl, 80 mM NaCl, 2 mM ethylene glycol bis-(2-aminoethylether) tetraacetic acid (EGTA), 50 mM Tris–HCl (pH 7.5), 0.05% NP-40, 1 mM MgCl_2_, 2 mM dithiothreitol, 10% glycerol and RNase Inhibitor (Roche) [18]. Samples were preincubated at room temperature with 50 µg of carrier *E. coli* tRNA for 20 min. The labelled RNA was then added and incubation proceeded for another 20 min. Samples were analysed by polyacrylamide gel electrophoresis and electroblotted to GeneScreen Plus membrane (NEN) using 0.5X tris-borate-EDTA (TBE) buffer. Signals were detected using a DIG Luminescent Detection Kit (Roche). Determination of the affinity of Mmi1 for the DSR core motif was done according to Zhang *et al*. [14]. In brief, EMSA was performed as described above, with 0.4 nM of DIG-labelled RNA carrying the *GFP* ORF, and four copies of the DSR core motif and 5–400 nM of GST-Mmi1. Bound and free RNA were quantified using LAS-1000plus (GE Healthcare). The *K*_d_ was calculated from the plot ln[RNA_bound_]/[RNA_free_] versus ln[protein_free_].

### 5.5. RNA-immunoprecipitation

Detailed conditions for RNA-IP are described by Hiriart *et al*. [29]. Immunoprecipitated RNA was isolated by phenol–chloroform extraction and reverse-transcribed according to the manufacturer's instructions (Superscript II, Invitrogen). Quantitative analysis of the relative RNA levels was performed using real-time PCR (Roche) and the MESA Green qPCR Master mix (Eurogentec). A pre-amplification step, done as described [30,31], was implemented owing to the low abundance of *sme2* RNAs using the following programme: 15 min incubation at 95°C, followed by 14 cycles of 95°C for 15 s, 60°C for 30 s and 70°C for 30 s. Four microlitres of a 1/400 final dilution of the preamplification reaction were used for the second quantitative PCR with a programme of 15 min incubation at 95°C, and followed by 40 cycles of 95°C for 15 s, 60°C for 30 s and 70°C for 30 s. Primers used are: *tub1* forward (5′-GTACTGGCCCATACCGTGAT-3′), *tub1* reverse (5′-CGAATGGAAGACGAGAAAGC-3′), *mei4* forward (5′-AAAAGCGACCTTCAAGCAAA-3′), *mei4* reverse (5′-TTGCATCGTTTGAGACTTCG-3′), meiRNA forward (5′-TGGTCATTCAAAAAGCTGGA-3′) and meiRNA reverse (5′-CTTGGGGGTTGGTTTAACTG-3′).

## Supplementary Material

Supplementary Figures
