# Supplementary material for: Hexanucleotide motifs mediate recruitment of the RNA elimination machinery to silent meiotic genes
Source: Open Biol. 2012 Mar;2(3):120014. doi: 10.1098/rsob.120014 (PMC3352096; doi:10.1098/rsob.120014)
Supplement: Supplementary Figures [file rsob120014-s1.pdf]

Figure S1

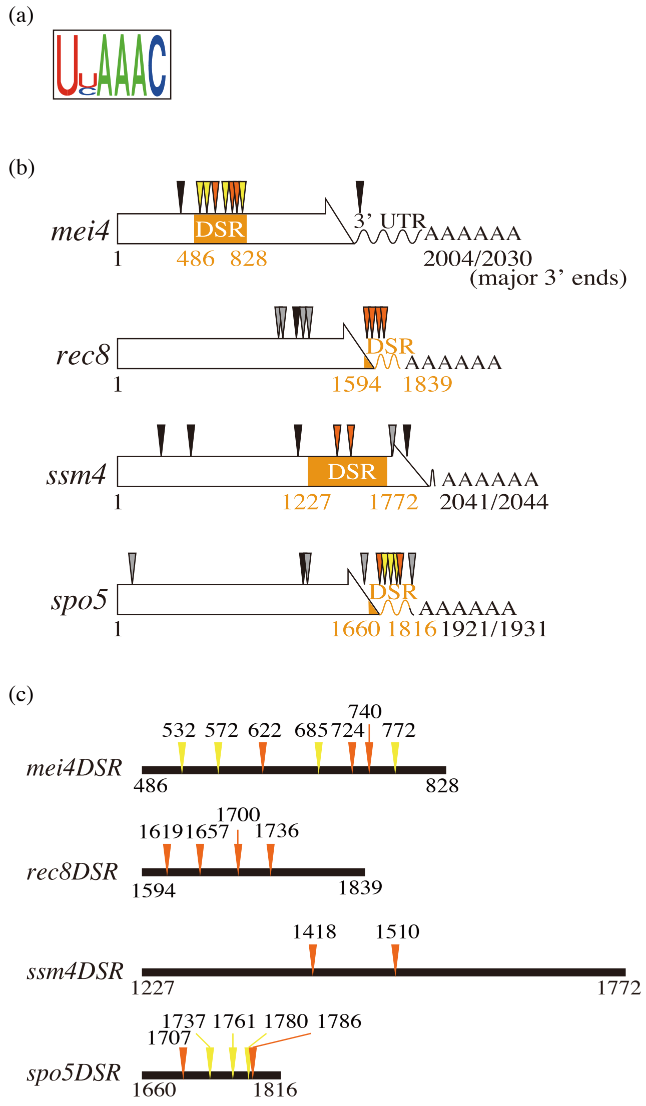

Figure S1. Consensus motif sequences in the DSR.
(**a**) The DSR core motif was determined by motif sampling analysis. (**b**) Alignment of the DSR core motifs from the *mei4*, *rec8*, *ssm4* and *spo5* mRNAs. The UUAAAC sequence is indicated by an orange arrowhead when it is within the DSR region and by a black arrowhead when outside. UCAAAC is indicated by a yellow arrowhead when it is within the DSR region and by a gray arrowhead when outside. The numbering of the nucleotides starts from each initiation codon. The precise 3' end(s) of each transcript was determined by RT-PCR. The results shown in the panel indicate that the distance between a DSR and the 3' end of the transcript is fairly variable, ranging from less than 100 to more than 1000 bases. (**c**) Precise location of the core motif within each DSR. Indicators are as described in (b).

Figure S2

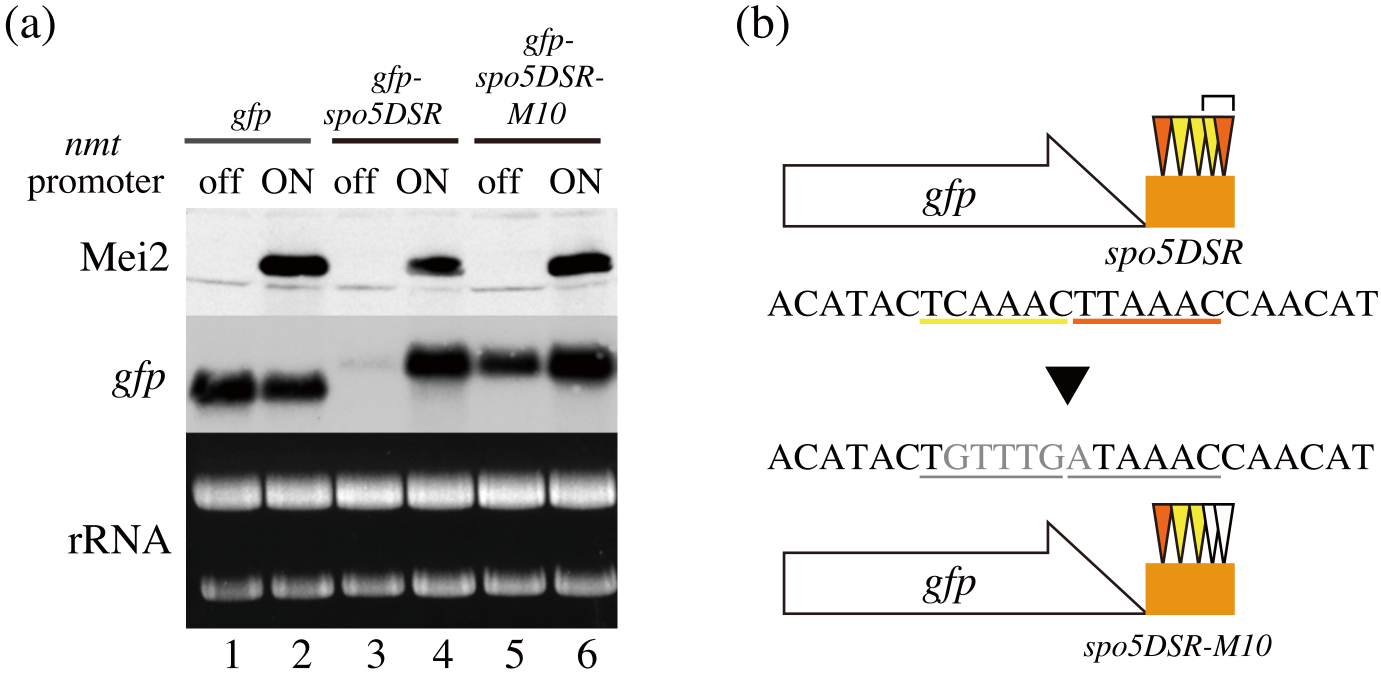

Figure S2. Loss-of-function mutation in the *spo5* DSR region.
(**a**) Expression of *GFP* mRNA from either pAGT1 (lanes 1 and 2), pAGT1-*spo5DSR* (lanes 3 and 4), or pAGT1-*spo5DSR-M10* (lanes 5 and 6) in JX383 cells (*nmt41-mei2-SATA*). Odd-numbered lanes represent cells growing mitotically, whereas even-numbered lanes represent cells undergoing Mei2-SATA-induced meiosis. Production of Mei2-SATA was detected by western blotting. rRNAs stained with ethidium bromide are shown in the bottom panel as loading controls. (**b**) Schematic illustration of the reporter constructs carrying *spo5DSR* (top) or *spo5DSR-M10* (bottom). Two core motifs are mutated in *M10*. Altered nucleotides in *M10* are shown in gray.

Figure S3

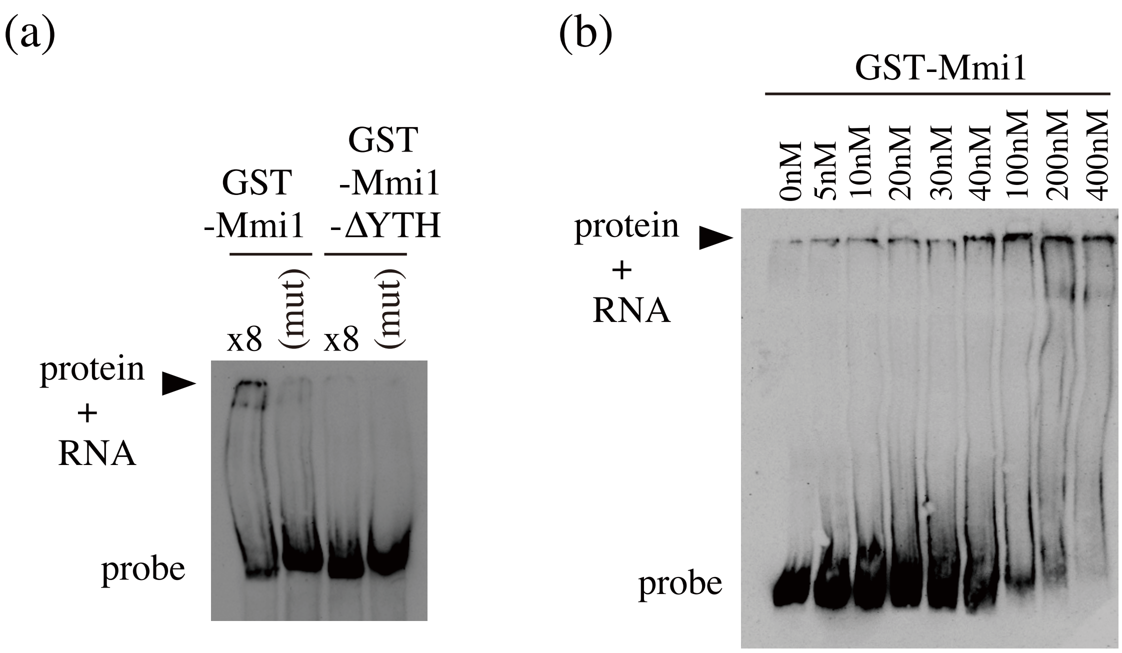

Figure S3. Mmi1 specifically binds to the DSR core motif *in vitro*.
(**a**) Electrophoretic mobility shift assay (EMSA) for the binding of GST-Mmi1 to eight tandem repeats of either the DSR core motif (UUAAAC) or a mutant form (GUAAAC) fused to the *GFP* ORF transcript. (**b**) Quantitative EMSA for the binding of varying concentrations of GST-Mmi1 to four tandem repeats of the DSR core motif (UUAAAC) fused to the *GFP* ORF transcript.

Figure S4

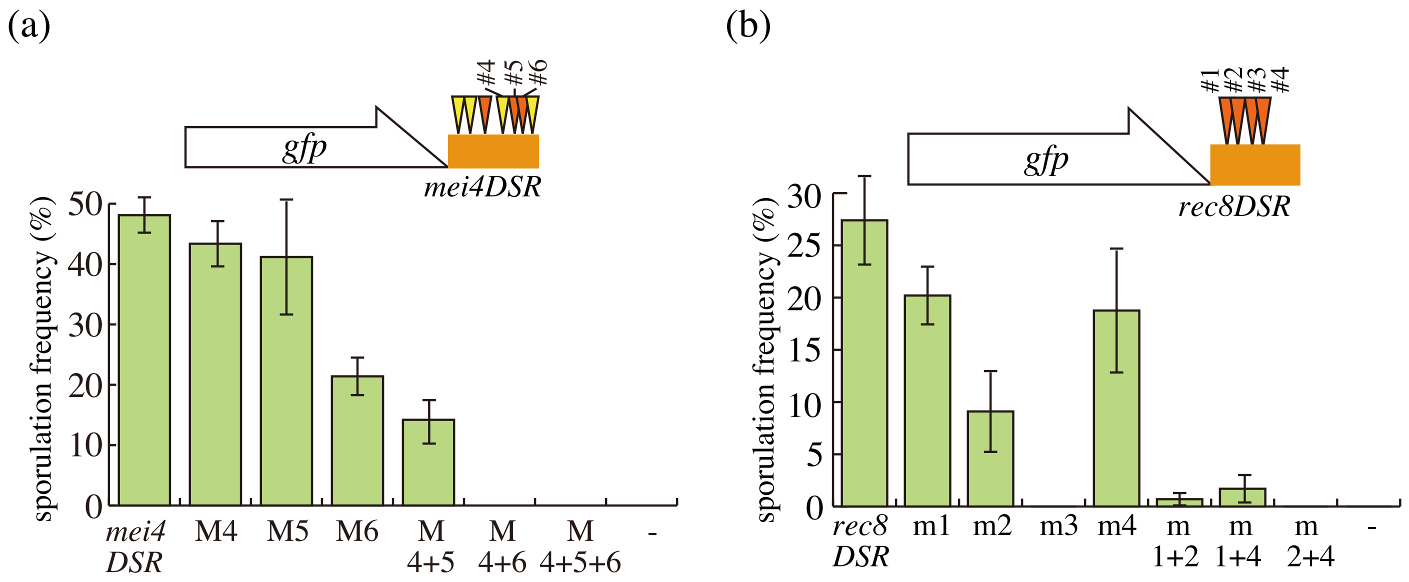

Figure S4. Dissection of the DSR region necessary to suppress *sme2Δ*.

(**a**) Sporulation frequency of JZ464 (*sme2Δ*) harboring pRGT1-*mei4DSR* or a mutant form of it. The *mei4DSR* mutants examined here carried an alteration in one of the core motifs #4, #5 or #6, or a combination of them. The control strain carrying pRGT1 is indicated by (-). Sporulation frequency was determined by microscopic observation of cells incubated on SSA medium at 30˚C for 3 days. Error bars indicate standard deviations (three measurements for each; total n>400). (**b**) Sporulation frequency of JZ464 cells harboring pRGT1-*rec8DSR* or a mutant form of it. The core motifs #1 through #4 in the *rec8* DSR were altered, and analysis was done similarly to (a).
